# Evaluating User Perceptions of Mobile Medication Management Applications With Older Adults: A Usability Study

**DOI:** 10.2196/mhealth.3048

**Published:** 2014-03-14

**Authors:** Kelly Anne Grindrod, Melissa Li, Allison Gates

**Affiliations:** ^1^School of PharmacyFaculty of ScienceUniversity of WaterlooWaterloo, ONCanada; ^2^School of Public Health & Health SystemsFaculty of Applied Health SciencesUniversity of WaterlooWaterloo, ONCanada

**Keywords:** medication therapy management, medication adherence, mHealth, mobile health

## Abstract

**Background:**

Medication nonadherence has a significant impact on the health and wellbeing of individuals with chronic disease. Several mobile medication management applications are available to help users track, remember, and read about their medication therapy.

**Objective:**

The objective of this study was to explore the usability and usefulness of existing medication management applications for older adults.

**Methods:**

We recruited 35 participants aged 50 and over to participate in a 2-hour usability session. The average age ranged from 52-78 years (mean 67 years) and 71% (25/35) of participants were female. Each participant was provided with an iPad loaded with four medication management applications: MyMedRec, DrugHub, Pillboxie, and PocketPharmacist. These applications were evaluated using the 10 item System Usability Scale (SUS) and visual analog scale. An investigator-moderated 30-minute discussion followed, and was recorded. We used a grounded theory (GT) approach to analyze qualitative data.

**Results:**

When assessing mobile medication management applications, participants struggled to think of a need for the applications in their own lives. Many were satisfied with their current management system and proposed future use only if cognition and health declined. Most participants felt capable of using the applications after a period of time and training, but were frustrated by their initial experiences with the applications. The early experiences of participants highlighted the benefits of linear navigation and clear wording (eg, “undo” vs “cancel”) when designing for older users. While there was no order effect, participants attributed their poor performance to the order in which they tried the applications. They also described being a part of a technology generation that did not encounter the computer until adulthood. Of the four applications, PocketPharmacist was found to be the least usable with a score of 42/100 (*P*<.0001) though it offered a drug interaction feature that was among the favorite features of participants. The usability scores for MyMedRec (56/100), DrugHub (57/100), and Pillboxie (52/100) were not significantly different and participants preferred MyMedRec and DrugHub for their simple, linear interfaces.

**Conclusions:**

With training, adults aged 50 and over can be capable and interested in using mHealth applications for their medication management. However, in order to adopt such technology, they must find a need that their current medication management system cannot fill. Interface diversity and multimodal reminder methods should be considered to increase usability for older adults. Lastly, regulation or the involvement of older adults in development may help to alleviate generation bias and mistrust for applications.

## Introduction

### Medication Adherence

As many as half of all prescriptions are not taken as prescribed, costing the US health system over US $100 billion per year [1-8]. A decade ago, the World Health Organization declared medication nonadherence to be “a worldwide problem of striking magnitude” and anticipated that “increasing the effectiveness of adherence interventions may have a far greater impact on the health of the population than any improvement in specific medical treatments” [7].

For chronic conditions such as diabetes, hypertension, and dyslipidemia, up to one-half of individuals will stop taking a medication as prescribed within the first year [8-10]. For psychiatric conditions, such as depression or bipolar disorder, one-half are nonadherent by 3 to 6 months [11,12]. The ability or desire to adhere is dependent on the duration of illness, the perceived benefit of therapy, adverse effects (real or potential), and the complexity of the regimen [13]. Effective interventions for nonadherence can be as simple as blister packing medications [13]. However, more intensive interventions are often needed to improve clinical outcomes, including patient education [11,13], team-based case management [13], patient self-management [14], telephone follow-ups [11], motivational interviewing, and provider support through pharmacist medication reviews or telephone reminders [11].

The language of adherence is complex and evolving. Adherence generally refers to how a patient takes a medication in relation to the prescribed timing, dose, frequency, and duration of therapy [13,15]. True adherence is difficult to measure but is often assessed through pill counting, prescription refills rates, and patient questionnaires [16]. Medication persistence is more specific and refers to how long a patient continues to take their prescribed therapy after it is first written [9].

The term “compliance” is often used interchangeably with adherence, but has fallen out of favor in recent years for its paternalistic implication that a good patient passively follows physician instructions [16]. “Concordance” has emerged as a more patient-centered term for adherence in the setting of shared decision-making, though a recent review found little agreement on the definition and scope of concordance and little evidence to support the value of concordant relationships [17]. For the purposes of this paper, we have chosen the term medication adherence to refer to the product of collaboration between a patient and a health care provider, wherein both collectively identify the goals of therapy and the therapeutic regimen [18].

### mHealth and Medication Therapy

Mobile health, or mHealth, applications offer one potential solution to help patients adhere to prescribed therapy. Over one-half of American adults own a smartphone and over one-third own a tablet [19,20]. One in 5 smartphone owners have downloaded at least one mHealth application [21]. Adults over age 50 are also accessing mHealth in increasing numbers. A 2010 survey by the American Association of Retired Persons showed that 89% of individuals over age 50 use a mobile device with the most common device being a cellphone and 7% using a smartphone [22]. Though 1 in 10 respondents are using an mHealth application to track health measures (eg, weight, blood pressure, blood glucose), 4 in 10 are interested in using one in the future [22].

For mHealth interventions to be both effective and accepted by end users, it is important to understand the differences between individuals who have, who will, and who will never adopt mHealth interventions. In the same way that there are barriers to medication adherence, there are also likely to be barriers to digital adherence. For example, user experience research has found users over age 50 can be easily frustrated by conceptual misunderstandings about the design of mobile devices [23,24]. They may also require training prior to use or may require devices tailored to their specific needs [25].

Among the thousands of mHealth applications commercially available, many are designed to help individuals organize and manage how they take their medications. Adults over age 50 make up the majority of medication users [26] and medication management applications need to consider this large target demographic. Our objective was to explore the usability and usefulness of existing medication management applications for adults over age 50.

## Methods

### Design and Setting

We used a mixed-methods approach to examine the usability and user perceptions of commercially available mobile medication management applications at the University of Waterloo School of Pharmacy. We included a qualitative assessment of user experiences using a grounded theory (GT) approach, which is reported according to the consolidated criteria for reporting qualitative research (COREQ) [27]. We also compared the usability of the test applications with the Systems Usability Scale (SUS), a validated measure of learnability and user satisfaction [28]. We did not develop any of the applications tested, and received ethics approval from the University of Waterloo Office of Research Ethics.

### Medication Management Applications

**Figure 1:**
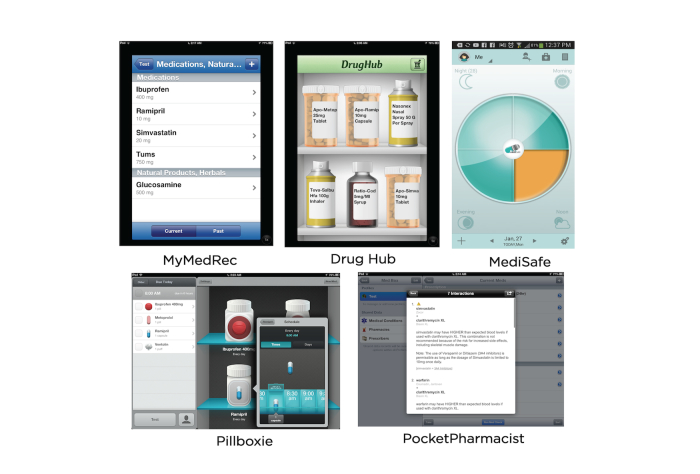
Screenshots of the mobile medication management applications included in the assessment.

We identified medication management applications by searching the Apples iTunes store using the terms “medication”, “prescription”, and “drug”. After exploring the descriptions of over 100 mobile applications, two researchers identified and downloaded 22 applications focused on helping consumers manage general medication therapy rather than medications related to a specific illness. The researchers independently explored the functionality of each application by entering a list of prescription and nonprescription medications, setting reminders, and reviewing the applications with friends and family members over age 50. After 2 weeks, the research team reconvened and chose five applications for the final review, each highlighting a different feature, such as appearance, reminders, drug information, drug interactions, and connectivity (Table 1, Figure 1).

We chose MyMedRec (Version 1.0.4) for its simple features and linear data entry. MyMedRec was developed as a collaboration between the Institute of Safe Medication Practices Canada, Canada’s Research Based Pharmaceutical Companies (Rx&D), and several health professional association across Canada. We chose Pillboxie (Version 2.6) for its graphical interface. A registered nurse in the United States developed Pillboxie to be a virtual medicine cabinet. We chose DrugHub (Version 1.3) for its drug information feature. The Great-West Life Assurance Company, a large provider of health insurance in Canada, developed DrugHub as a service to the general public. We chose PocketPharmacist (Version 3.1.8, Danike, Inc.) for its drug interaction feature. A pharmacist in the United States designed PocketPharmacist to provide users with medication information and the ability to check multiple drugs for any interaction. Finally, we chose MediSafe (Version 2.3.2, MediSafe Project) for its cloud-synced, family-centered profile sharing features. MediSafe was designed in Israel. At the time of the study, MyMedRec, Pillboxie, DrugHub, and Pocket Pharmacist were available for the iOS system, and Pocket Pharmacist and MediSafe were available for the Android OS.

**Table 1:** Features of the mobile medication management applications selected for review.

Full name	MyMedRec	Pillboxie	DrugHub	Pocket Pharmacist	MediSafe
Medication list	✓	✓	✓	✓	✓
Reminder alarms	✓	✓	✓	✓	✓
Drug information			✓	✓	
Drug interactions				✓	
Multiple user profiles	✓	✓		✓	✓
Profile sharing via email	✓	✓	✓	✓	✓
Sharing across multiple devices					✓

### User Testing

#### Participants and Sampling Frame

We included participants aged 50 years of age and over, who could speak and read English and who took some form of chronic medication. We did not require participants to have previous experience using a touchscreen device. We recruited participants by posting flyers and attending events at community centers and medical clinics. Our sampling strategy reflected a GT approach to qualitative analysis [29]. As we did not have any preconceived theories about the usability or usefulness of the applications for older adults, we began by purposively recruiting a sample of 13 participants who had a range of experience using medications and touchscreen devices [29]. We then recruited a theoretical sample of 22 participants to expand on the theories framed from the purposive sample [29]. According to Glaser [30] for the theoretical sample, “the analyst jointly collects, codes, and analyzes his data and decides what data to collect next and where to find them, in order to develop his theory as it emerges.” As described by Draucker et al [31], we used several strategies for identifying the theoretical sample, including intensity sampling of individuals who would have “a lot to say” (eg, information technology experts), typical sampling of average users, extreme case sampling (eg, individuals with complex health conditions), and purposeful sampling to represent subgroups (eg, married couples). We continued sampling until data saturation was reached.

Our final sample included 35 participants aged 52-78 years (mean 67 years), 71% (25/35) of whom were female (Table 2). All but 2 participants reported at least one chronic medical condition and all participants were taking at least one medication, including prescription products, vitamins, and natural health products. Our sample included a range of low- to high-income participants, almost one-half of whom had a post-secondary degree. Most participants used a computer daily and over one-third used a tablet or smartphone daily.

#### Procedure

**Figure 2:**
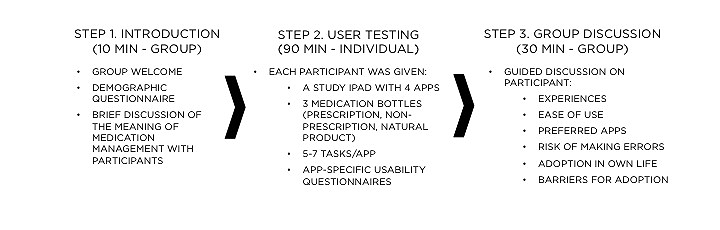
Study design.

We used a group-based assessment model to meet our objectives as it allowed us to test multiple participants at once while capturing moments of consensus, censoring, and dissonance among new, novice, and experienced mobile device users (Figure 2) [32]. The number of study devices we had access to limited our group size to a maximum of 8 participants. Our final groups included between 3 and 7 participants.

We began each session with a meal or light refreshments and a 10-minute discussion of what medication management meant to participants. Each participant was provided with a third generation Apple iPad and given a series of ordered tasks. Participants in the purposive sample worked from the simplest application to the most complex (MyMedRec, Pillboxie, DrugHub, PocketPharmacist). For the theoretical sample, we randomized the order of the applications to assess order effect. We also introduced styluses and gave participants access to smaller devices such as the Apple iPhone 4, Apple iPod Touch, and the Samsung Galaxy S3. We introduced the fifth application, MediSafe (Medisafe Project LTD), because it had a unique pillbox graphical interface and had more connectivity than the other study applications, but excluded it after one session because participants complained of several system errors.

For each application, we asked participants to complete a series of application-specific tasks that could include the following: (1) adding prescription, nonprescription, and natural medicines, (2) scheduling reminders, (3) recording when a dose was taken, (4) emailing profiles, (5) reading drug information, and (6) scanning for drug interactions. We provided each participant with a set of standardized medication bottles that represented a clinically significant drug-drug interaction: warfarin, aspirin, and St. John’s Wort; clarithromycin and atorvastatin; ramipril and ibuprofen; furosemide and ibuprofen; and levothyroxine and calcium carbonate [33]. We provided limited assistance to users who failed to complete a task after several attempts.

We concluded each session with a 30-minute focus-group discussion. The guided discussion included questions on overall user experiences, ease of use, concerns over the potential for data input errors, perceived quality of the information provided, preferences for different features, and expected adoption by adults over age 50. On completion, participants were given a $10 gift card in appreciation for their time.

**Table 2:** Participant characteristics (N=35).

Characteristic	Category	n (%)
**Age**	Median (range)	67 (52-78)
**Gender**		
	Male	10 (29)
	Female	25 (71)
**Medical Condition(s)**		
	None	4 (11)
	Heart disease	7 (20)
	Cholesterol	14 (40)
	High blood pressure	15 (43)
	Thyroid disease	5 (14)
	Bone and joint problems	6 (17)
	Cancer	2 (6)
	Diabetes	7 (20)
	Kidney disease	4 (11)
	Liver disease	0 (0)
	Lung disease	2 (6)
	Other	10 (29)
**Medications**		
	Prescription medications	30 (86)
	Vitamins	28 (80)
	Natural health products	16 (46)
	Manage medications for others	14 (40)
**Highest level of education**		
	High school	9 (26)
	College	13 (37)
	University	7 (20)
	Graduate degree	6 (17)
**Annual household income**		
	< $20,000	2 (6)
	$20,000 - $49,999	10 (29)
	$50,000 - $79,999	10 (29)
	>$80,000	6 (17)
	Prefer not to say	7 (20)
**Use a computer**		
	Daily	27 (77)
	Weekly	3 (9)
	Monthly	0 (0)
	Rarely	1 (3)
**Use a tablet**		
	Daily	10 (20)
	Weekly	1 (3)
	Monthly	0 (0)
	Rarely	0 (0)
**Use a smartphone**		
	Daily	5 (14)
	Weekly	3 (9)
	Monthly	0 (0)
	Rarely	0 (0)

### Data Collection and Analysis

Participant demographics and experiences were gathered using paper-based questionnaires and summarized using descriptive statistics. Participants were encouraged to write comments about their experience as they tried each application. Two researchers also recorded their observations of participant experiences, including questions asked, errors observed, and tasks users could not complete. Focus-group discussions were audio recorded. All data, including audio recordings, field notes, and participant comments, were transcribed and de-identified by a researcher and double-checked by a second researcher.

After each application, participants rated usability on a visual analogue scale (easy/difficult) and the 10-item SUS (Multimedia Appendix 1) [28]. The SUS is a validated tool that uses a 5-point Likert scale to provide a quantitative measure of the usability and learnability of a system and provide an overall score between 0 and 100 [28]. SUS scores were analyzed in SPSS using a repeated-measures analysis with post hoc tests to determine where usability differences occurred.

### Grounded Theory Analysis

We used a GT approach as it allowed us to develop a theoretical interpretation of the meanings we observed older users attaching to mobile medication management applications without defining the phenomenon a priori [34]. For GT, data are systematically collected from many sources, including participant experiences, both written and voiced, and researcher observations. GT is a constant analysis method where data and emergent theories are continuously compared to ensure resulting theories are grounded in raw data. We based our analysis on the descriptions of GT by Patton [29], Glaser [30], Strauss and Corbin [35], and Suddaby [36].

Our data analysis, which began after the third session, followed three levels. For the first level of analysis (microanalysis), two independent researchers coded data by briefly summarizing each line of data. In the second level of analysis (axial coding), the two researchers organized the summaries into categories (Table 3). To assess interrater reliability, a third researcher compared the codes and categorizations for the first three sessions and any disagreements were resolved by discussion. For the third level of analysis (theory development), the emergent theories were formulated into a “logical, systematic and explanatory scheme” by the three researchers [35]. To minimize our own biases, we reflexively reviewed the transcripts a final time to identify any supporting quotations and contradictory data [37].

**Table 3:** Categories and labels used to organize grounded theory analysis.

Category	Examples of descriptive summaries
What does “medication management” mean?	-Remembering medications -Understanding medications -Drug–drug, drug–food interactions -Relationship with physician -Pill boxes -Renewing prescription
How did it feel to try the applications?	-Feelings: frustrating, challenging -Positive learning -Uncertainty of future adoption -Information overload -Shift from frustrating to doable
What was the easiest/most difficult application to use?	-Training would help make it easier -Prior experience required -Gender distinctions -MyMedRec, because it was easier to find way around -PocketPharmacist had too many submenus
What was the most/least preferred application?	-Not qualified to evaluate -Lack of experience with technology -Liked different features from each -Drug information applications -Application glitches
What features were liked?	-Graphics easier to understand than words -High value for listing supplements -Drug interactions -The simpler the better
What features were disliked?	-Ambiguous use of symbols -Requires previous knowledge of device -Not intuitive to look at corners -Unreliable reminders -Lack of food–drug interaction -Microscopic keyboard
What features were most surprising?	-Missing allergy–drug alerts -Untrustworthy drug interaction feature for PocketPharmacist -Lack of features on many apps -Surprised there was an email feature
How can/will the applications be used in real life?	-Uncertain if would ever use -Younger person associated with technology -Possible user: elderly relative, someone with 8 medications/day -Way of the future -Current management system works
What is the willingness to pay for the applications?	-Willing to use more a difficult application if free -Uncertain -Need a trial period -Surprised by the price, seems low -$100 for PocketPharmacist
How long would someone spend learning the applications?	-Willingness to spend time everyday -It would take a week to learn with daily use -Overall it was not intuitive -Might spend a couple hours -I would give it 15 minutes
Should physicians or pharmacists recommend the applications?	-All younger persons already own technology -Not applicable to current older adults -Older generation is adopting new technology -Pharmacist job to educate patients on drugs -Variable levels of pharmacists -Providers are intolerant of nonadherent patients
Should the applications connect with physician or pharmacist computer systems?	-Future connection “so cool” -Useless if increased workload -Intolerance to uncooperative providers -No, my doctor will not accept email -Prefer to talk to the pharmacist or doctor in person
Should the applications be backed up?	-Expect backing up -Assume data is retrievable -Expect it to be saving something -Fact that it’s not backed up takes away from the usefulness -Thought the iPad could be backed up to the laptop

## Results

### User Testing

Based on the SUS scores (Table 4), the *F* test indicates PocketPharmacist had a significantly lower usability score compared with all other applications (*P*<.0001), whereas MyMedRec, Pillboxie, and DrugHub were not significantly different from each other. Order of use did not affect SUS scores when added to the model (*P*=.44).

**Table 4:** Overall Systems Usability Scale scores for each application assessed.

Application	Mean SUS score (SD)^a^
DrugHub (N=35)	57.1 (22.2)
MyMedRec (N=35)	55.6 (22.4)
Pillboxie (n=31)	52.2 (18.1)
PocketPharmacist (N=35)	42.1 (18.7)
MediSafe (n=4)	40.0 (15.1)

^a^Significant difference between applications *f*=2.95, *P*<.0001

### Grounded Theory Analysis

**Figure 3:**
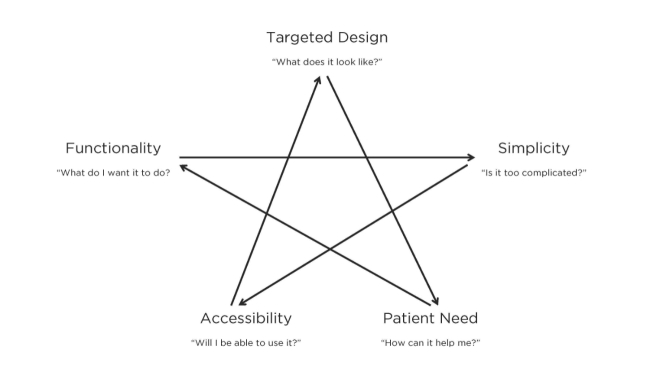
Model depicting first time experiences of older adults using mobile medication management applications.

Figure 3 describes the theoretical construct that emerged in our analysis, including the interplay between usability, accessibility, design, and need.

### Targeted Design

For participants, the early experience of learning to use a mobile medication management application was frustrating, overwhelming, and challenging but it was also fascinating, fun, and enlightening (Table 5). As one participant noted,

With a little bit of practice, with all of them, [it] would become a lot easier to like anything. You know, the first try at any of these, regardless whether you’re familiar with an iPod or an iPad, it does not go smoothly. They’re not terribly intuitive.

Male, Group 8

Early on, we observed that many participants were reluctant to learn by trial and error. They appeared to feel vulnerable or lost and often worried about making a mistake. Many spoke of trying not to break the device. We learned to start each session saying, “Don’t worry, you won’t break it” to encourage learning by trial and error. In describing the need for support, one participant explained,

there are a few steps missing I think in each of these [applications]…I didn’t know how to get from a certain screen to another, it wasn’t very evident, but once I was shown, I think it’s easy to use.

Female, Group 3

Eventually, within the 2-hour timeframe of each session, almost all participants became comfortable inputting information. The experience of learning the applications can be compared with the implementation of new technologies in other areas of life, including banking and transportation:

I guess while I was doing it, I kept thinking about ok, when I was first ordering airline tickets on the computer, or bus tickets on the computer, the difference after you’ve done it a few times as opposed to the first time, trying to figure out which buttons to hit.

Female, Group 1

However, some individuals had more difficulty than others, and would likely need significant support, both technical and emotional, to adopt the application into their lives. One participant who found the applications extremely difficult to use highlighted this challenge when he concluded,

I don’t want an app, I don’t ever want to see one in my house or anything because to me they’re just frustrating. Frustration devices.

Male, Group 5

When asked if they were willing to persist in using the applications, the opinion was divided between willingness to persevere until comfortable given a perceivable benefit, or only for 15 minutes due to impatience and lack of time.

I’d spend a bit of time, half an hour to an hour, not even an hour, half an hour to learn something like this, yes.”

Female, Group 6

Patient Need

The perceived usefulness of a mobile medication management application is closely related to the needs of the end user. Users who believed their current adherence strategies are sufficient are likely to consider the applications as less useful. One participant highlighted the importance of perceived usefulness by saying*,*

I like things fast and dirty, I would just give up. Say forget it, I’ll just do it my old fashioned way.

Male, Group 6

Strategies for remembering to take medications included scheduling all medication doses at once, using a physical pillbox, wearing a digital watch with an alarm, and/or having a pharmacist who provides telephone refill reminders. Many also carried a written or printed list of medications in a wallet or purse. Participants would only speculate future use under the assumption of declining health, declining memory, or the need to manage medications for a relative.

I’m looking at it from the point of view of my mother when she was elderly. She was confined to a wheelchair and okay, she wasn’t computer literate. But had she been, you know, if she’d had it with her, in her chair, she could’ve looked at it and said ‘yeah hey I need to take this pill’ or there’s a reminder, or ‘no I can’t’, somebody’s making lunch for her, ‘no I can’t have grapefruit because I’ve taken Lipitor’ or something like that. I’m sure that kind of information would have been good. If you’re not terribly mobile, I think something like that, and in this day and age, as time goes on, people are much more computer literate and can handle these things much more easily and how do you say, you know, that would be much more useful, if you’re concerned, if you’re taking a lot of medications. Because I know some people who are taking seven or eight a day.

Female, Group 1

Most also felt it was only appropriate for health care professionals to recommend an mHealth application if they had used it themselves and if the patient was willing, needed it, and was technologically literate.

**Table 5:** Participants summarize the experience of trying mobile medication management applications in one word.

Negative Words (Count)	Neutral Words (Count)	Positive Words (Count)
Frustrating (5)	Different (1)	Fascinating (2)
Challenging (3)	Perplexing (1)	Fun (2)
Overwhelming (2)		Enlightening (1)
Stressful/nerve-wracking (2)		Doable (1)
Confusing (1)		Interesting (1)
Exhausting (1)		Useful (1)
Complicated (1)		Informative (1)

### Functionality

Mobile medication management applications can be conceptually separated into two categories: adherence (MyMedRec, Pillboxie) and information (DrugHub, PocketPharmacist). An “ideal" application was described as including both features:

I found MyMedRec covers everything, it’s ordered properly. But it did miss the other little features, the little pillbox [in Pillboxie] and then the [drug interaction] check [in Pocket Pharmacist] and then [DrugHub]. I guess it’s the access to the information and whether you could check interactions and things like that. If somehow you could incorporate that into the [MyMedRec] then it would be perfect.

Female, Group 3

In their daily lives, all participants sought information about their medications in order to stay aware and avoid adverse events. The drug information features were seen as providing background information on a new prescription, supplementing the information given by a health care professional, and satisfying curiosity.

For me, personally, I take a lot of pills everyday…I’ve got it so down and whenever I take a prescription, well the pharmacist is very good to go over things, but I always, always make a point of reading the literature when I get it.

Female, Group 2

While the most popular source of drug information was the pharmacist, some participants worried that too much information was dangerous and that the applications were replacing the expertise of the pharmacist. Given the choice, participants preferred an in-person conversation for important information:

Something like drug interactions? I don’t want to be bothered by anything like that. I mean I know I should, but I want my pharmacist to say to me when I go in, don’t take this or do take that. You know what I mean? I didn’t go to school, I don’t want to have the responsibility of worrying about that...

Female, Group 1

When asked to estimate the cost of applications, most participants valued drug information applications over adherence applications. Participants who had purchased applications expected to pay less than Can $5 (or often nothing at all), but those who had never purchased an application expected to pay up to Can $100 or a monthly fee. Most did not take into consideration the cost of the device itself.

### Simplicity

There was a competing relationship between functionality and complexity. The “ideal” application may actually be two applications, one for adherence and another for information. Separating the features into two applications would maximize the functionality of both features rather than trying to do both incompletely.

I think there’s two parts of it. There’s reminding people to take the medication but then there’s the whole information side with what’s working with what. So it almost seems like you should have two apps.

Female, Group 7

For many participants, linear navigation was preferred. Participants commonly struggled with going “back and forth”, essentially, moving forward to enter a medication into their profile and once completed, going back to enter a new medication (Table 6). Moving backwards also referred to fixing a mistake. Participants moved from one main menu and followed single pathways to perform or correct a task. As such, most found MyMedRec and DrugHub to be easier and more logical. In contrast, Pillboxie and PocketPharmacist did not flow because, rather than advancing through different screens, navigation was broken into submenus or different windows on a single screen. Inconsistencies also caused confusion. To return to a previous screen or menu, MyMedRec and DrugHub used the standard iOS arrow pointing to the left corner. To back out of a task in Pillboxie, users had to tap outside the task window.

I’m impatient as hell. So when it comes to an app, it’s got to be simple. See…it wasn’t easy for me to find the prompts, you know, partly from [my] glasses, but also I’m impatient and I quit looking. And I said ‘Oh I’ve spent all this time entering the stuff but if I put CANCEL, does that mean it’s gone?

Male, Group 5

Similarly, the first screen a user saw with PocketPharmacist contained both a menu and submenu. Participants expressed feeling overwhelmed by the amount of information being presented all at once. Many first time users struggled with basic touchscreen features, such as accessing and using the keyboard and employing application-specific gestures. One participant felt this should be standardized,

So ultimately, you’d want a universal language, and a universal kind of, you know, this is where the back button is, this is where the forward button is. But if that doesn’t happen, then every app has its own unique way.

Female, Group 2

**Table 6:** Application actions and features identified by participants as being nonintuitive or difficult to interpret.

Action/feature	Description of challenge
A “+” to add a new item	Though typically used to add a new item, the symbol had little meaning for first time users. Also, because it is often found in the top corners it is easily missed.
Go back	The back arrow is used to return the user to the previous screen but instead of testing the button, the research team was often asked, “How do I go back without losing my information?”
Cancel	The word “cancel” typically means “undo” but many participants felt it implied finality and described how they “cancel” social or service contracts such as memberships, subscriptions, and appointments.
Scrolling	Without a scrollbar, participants rarely looked for additional information.
Audio reminders	The audio alarms were inaudible to many participants, especially males.
Autocorrect	When typing, many participants focused on the keyboard and missed the autocorrect feature that would change drug names or dosage units (eg, “mcg” to “mg”).
Inconsistent terminology	Inconsistent terminology led participants miss features. For example, reminder features were called “schedule,” “dose reminder,” or “first dose” in each application.
Sample text	Greyed text was used to provide examples of data that could be entered into a field, but participants typically misinterpreted the grey text to be the information of another user.
Peripheral buttons	Participants associated a black frame as being outside of the application and noninteractive, thus overlooking peripheral buttons completely.

### Accessibility

One of the challenges faced by participants was that the adherence features we examined (dose reminders, refill reminders) made assumptions about the end user. For example, the reminder strategies (alarms, notification boxes) assumed users were “attached” to mobile devices. Participants said, for example,

Like, [young people] live with their cell, live with their Blackberry, and that becomes, you know what I mean. Like, I could see, even obviously, when those kids get to be 50, they will still be attached to the hip with those Blackberries.

Female, Group 1

Comparing the use of the applications on the tablet to the smartphone, one participant noted,

Reminders would probably be the best [feature] but it would be inconvenient unless I had one of the other devices that you could carry in your pocket or your shirt pocket or a woman could carry in her purse. (Male, Group 4).

This is an important distinction because though the tablets are less portable, they are more accessible to individuals with age-related vision loss. In one case, a participant with severe low vision noted that touchscreen devices were surprisingly accessible,

I was always afraid to even look at them or try them, because I just thought that I wouldn’t be able to see, so why even bother. But I was surprised…yeah.

Female, Group 4, low vision

The participants, as older adults, also described how they power off devices between use to conserve battery power or save it for emergencies.

These tablet things, they’re not plugged in, so, most of the time…you tend to turn them off to conserve the battery and maybe they could be designed so that they automatically turn themselves on, give a signal, and then go back to rest.

Male, Group 8

The reminder strategies also assumed users were physically able to hear alerts. In every session, we observed at least 1 participant, often male, who could not hear the alarms going off in the room.

But some people may be hearing impaired and you know, maybe that could be accommodated, I’m not sure how. [Male, Group 8].

Finally, participants worried that adherence strategies that required users to maintain a medication administration record were easily fallible.

With your daily plastic, you can see that you took it, with this thing, you may have put in that you took two pills…but you got distracted, so how do you know you took two?…It’s not physical, you can’t see it, as you get older. I mean, there’s two sides. You forget to take things or you know, things like that, I don’t know, it’s too easy to screw up.

Female, Group 6

## Discussion

### Overview

We asked 35 adults aged 50 and over to spend 2 hours trying popular medication management applications on mobile devices. Most were new and novice users and we found that the currently available applications were not designed with older users in mind. Without simple and targeted design, the current applications are unlikely to be considered useful, usable, or accessible for a large proportion of individuals who need to take chronic medications.

### Acceptance of Mobile Medication Management Applications

Dr Everett Koop famously said, “Drugs don’t work in patients who don’t take them”. Similarly, mobile medication management applications will not work for users who do not use them. Reminders offered the clearest example of the gap between design and the older end user. One-half of prescriptions are not taken as directed [2,6], and dose reminders have the potential to help individuals remember doses that would have otherwise been missed. Most reminder systems also provide a record of adherence that could be used to help participants or clinicians understand the impact of nonadherence on their disease. However, in our study, reminders were widely considered to be of little use to older adults, many of whom have age-related hearing loss or who prefer to either leave their mobile devices at home or power devices off between use.

The Technology Acceptance Model, which was introduced by Davis in 1986 [38], suggests that acceptance depends on a user’s perception of the usefulness and ease of use of a system. Similarly, the diffusion of innovation model emphasizes that a new technology needs to offer a “relative advantage” over the status quo [39]. The lack of perceived usefulness or relative advantage appeared to be the greatest barrier to acceptance or adoption in our study. Our participants felt capable of, and interested in, using mobile applications to manage medications, but they searched for a need that their current medication management system was not filling.

In a study of the factors that impact patient acceptance of online self-management technology, Or et al [40] concluded that the first step in designing a system that patients will accept is to focus on their needs. Barriers to medication adherence are complex and interwoven and not easily addressed by mobile applications [7-12]. In addition to age, barriers include health literacy, the health care provider-patient relationship, poor memory, care team size, medication efficacy, and the complexity of medication regimen [41]. Future mobile applications should be designed with the awareness that multimodal methods for nonadherence are more successful than interventions focusing on one aspect of nonadherence [11,13]. For example, the next generation of applications could provide users with access to plain language information on medications both before and after a prescription is filled, include accessible reminders for doses and refills, and offer strategies to simplify a dosing regimen. More advanced iterations could also help the consumer alert clinicians when they have a question, experience a side effect, or are unable to afford the cost of their prescribed therapy.

### Application Development for Older Adults

We were able to speak with 3 of the 5 creators of the medication management applications tested. None had conducted usability testing in adults 50 and older. A tendency to target designs toward younger adults perpetuates the notion of the “digital divide” or “digital disengagement”, where developers assume older adults lack the technological access and literacy of younger generation [42]. Loos [43] has cautioned against using age as the ultimate explanatory variable when examining the digital divide. By overlooking older users, developers may be failing to reach a major target demographic.

While we need to recognize that many older users are technologically savvy, Hawthorn [44] notes that we still need to make accommodations for “age-restricted users” with age-related changes in hearing, vision, cognition, and mobility. Application designers can consider the impact of age on usability and include large font, clear buttons, and high-contrast text. However, before developing an application for medication adherence for a specific operating system, the design team should consider the suitability of the mobile device for the target population. Most existing mobile devices should work well for addressing barriers such as lack of information. For younger people, they may also be useful for the day-to-day barriers to compliance such as forgetfulness. However, for older populations, forgetfulness and treatment complexity may be better suited to multimodal mobile interventions that include companion pillbox devices, for example.

To improve usability, it would be helpful for developers to provide clear instructions and describe important buttons or features for first time users (eg, “The area where you list all the medications you take is called the *Med Box*. Use this area to enter the names and doses of your medications”). Although some applications did provide examples, they were often small, low contrast, and easily misinterpreted as pre-entered information. The Nielsen Norman Group refers to the inability of identifying a touchable area as low discoverability [45]. To improve developers and designers should consider working with health organizations to identify target users who can participate in early- and late-stage usability testing.

Often, mHealth applications do not follow guidelines or include features considered essential for prevention and public health [46,47]. The lack of standardization and regulation may limit adoption by users over age 50 and their health care providers. For example, participants were suspicious of an application put forth by an insurance company. They were afraid personal information could be collected and used against their claims. On the other hand, when we spoke with the developers, no data were collected from the applications. Sentiments of use and privacy as barriers for adoption match with those from respondents in the 2010 AARP survey [22]. To promote consumer trust, one possibility is to develop a systematic self-certification model similar to the Health On the Net Foundation [48].

### Strengths and Limitations

A strength of our study is that it reflects the experiences of first-time users. The rationale was that, in the real-world setting, many new users would try several medication management applications before choosing one to use or would be prescribed a mobile application that they had not used previously. However, the 2-hour format meant some features of each application could not be explored. In some cases, participants assumed certain features were missing. In each case, we demonstrated the feature in question to gather any additional feedback. During the discussions, application names and features were often confused with one another so we tried to ensure we regularly revisited each application to clarify which application was being discussed.

In future studies, participants should be given a device for a longer time period to examine the effects of daily use. Previous studies have found SUS scores to increase with the degree of experience with a program prior to usability testing [49]. Longitudinal usability testing may help uncover long-term benefits and drawbacks that first-time experiences cannot. Alternatively, usability testing of medication management applications with health care professionals may provide a deeper understanding of their acceptance and willingness to provide additional services in relation to application use by patients. Another potential solution that should be further explored is the combination of an application with a wearable device such as a smartwatch or wristband.

### Conclusions

Though older adults make up the majority of medication users, mobile medication management applications are often designed for younger populations. The result is that age-related physical changes, such as hearing or vision loss, and the use of nonintuitive design features limit the usefulness of the applications for older users. When developing mobile interventions to improve medication use and adherence, designers, programmers, and developers need to consider older adults as potential high-impact end users and include this population in the design process. For older adults, standard features such as reminders may be poorly suited to most mobile devices, whereas applications that provide high quality information on side effects or drug interactions may be more desirable. Considering that the industry is not currently regulated, the focus also needs to be on building applications that limit the risk of errors and omissions.

## Multimedia Appendix 1

Systems Usability Scale used to compare selected medication management applications.

## Abbreviations

COREQ: consolidated criteria for reporting qualitative research
GT: grounded theory
SUS: Systems Usability Scale
