# Supplementary material for: Evaluating User Perceptions of Mobile Medication Management Applications With Older Adults: A Usability Study
Source: JMIR Mhealth Uhealth. 2014 Mar 14;2(1):e11. doi: 10.2196/mhealth.3048 (PMC4114457; doi:10.2196/mhealth.3048)
Supplement: Supplementary file 1 [file mhealth_v2i1e11_app1.pdf]

Multimedia Appendix 1

Table. Systems Usability Scale used to compare selected medication management applications.

		Strongly Disagree	Disagree	Neutral	Agree	Strongly Agree
1	I think that I would like to use [app] frequently.	1	2	3	4	5
2	I found [app] unnecessarily complex.	1	2	3	4	5
3	I thought [app] was easy to use.	1	2	3	4	5
4	I think that I would need the support of a technical person to be able to use [app].	1	2	3	4	5
5	I found the various functions in [app] were well integrated.	1	2	3	4	5
6	I thought there was too much inconsistency in [app].	1	2	3	4	5
7	I would imagine that most people would learn to use [app] very quickly.	1	2	3	4	5
8	I found [app] very cumbersome to use.	1	2	3	4	5
9	I felt very confident using [app].	1	2	3	4	5
10	I needed to learn a lot of things before I could get going with [app].	1	2	3	4	5
